# Immune-associated biomarkers identification for diagnosing carotid plaque progression with uremia through systematical bioinformatics and machine learning analysis

**DOI:** 10.1186/s40001-023-01043-4

**Published:** 2023-02-23

**Authors:** Chunjiang Liu, Liming Tang, Yue Zhou, Xiaoqi Tang, Gang Zhang, Qin Zhu, Yufei Zhou

**Affiliations:** 1 Department of General Surgery, Division of Vascular Surgery, Shaoxing People’s Hospital (Shaoxing Hospital of Zhejiang University), Shaoxing, 312000 China; 2 Department of Rehabilitation, The First Affiliated Hospital of Anhui Medical University, Anhui Public Health Clinical Center, Hefei, 230000 Anhui China; 3 Hepatobiliary CenterKey Laboratory of Liver TransplantationNHC Key Laboratory of Living Donor Liver Transplantation, The First Affiliated Hospital of Nanjing Medical UniversityChinese Academy of Medical SciencesNanjing Medical University), Nanjing, 210000 Jiangsu China; 4 Shanghai Medical College, Fudan University, Shanghai, 200032 China

**Keywords:** Unstable carotid plaque, Uremia, Diagnostic biomarker, Bioinformatics analysis, Machine learning, Immune cell infiltration

## Abstract

**Background:**

Uremia is one of the most challenging problems in medicine and an increasing public health issue worldwide. Patients with uremia suffer from accelerated atherosclerosis, and atherosclerosis progression may trigger plaque instability and clinical events. As a result, cardiovascular and cerebrovascular complications are more likely to occur. This study aimed to identify diagnostic biomarkers in uremic patients with unstable carotid plaques (USCPs).

**Methods:**

Four microarray datasets (GSE37171, GSE41571, GSE163154, and GSE28829) were downloaded from the NCBI Gene Expression Omnibus database. The Limma package was used to identify differentially expressed genes (DEGs) in uremia and USCP. Weighted gene co-expression network analysis (WGCNA) was used to determine the respective significant module genes associated with uremia and USCP. Moreover, a protein–protein interaction (PPI) network and three machine learning algorithms were applied to detect potential diagnostic genes. Subsequently, a nomogram and a receiver operating characteristic curve (ROC) were plotted to diagnose USCP with uremia. Finally, immune cell infiltrations were further analyzed.

**Results:**

Using the Limma package and WGCNA, the intersection of 2795 uremia-related DEGs and 1127 USCP-related DEGs yielded 99 uremia-related DEGs in USCP. 20 genes were selected as candidate hub genes via PPI network construction. Based on the intersection of genes from the three machine learning algorithms, three hub genes (FGR, LCP1, and C5AR1) were identified and used to establish a nomogram that displayed a high diagnostic performance (AUC: 0.989, 95% CI 0.971–1.000). Dysregulated immune cell infiltrations were observed in USCP, showing positive correlations with the three hub genes.

**Conclusion:**

The current study systematically identified three candidate hub genes (FGR, LCP1, and C5AR1) and established a nomogram to assist in diagnosing USCP with uremia using various bioinformatic analyses and machine learning algorithms. Herein, the findings provide a foothold for future studies on potential diagnostic candidate genes for USCP in uremic patients. Additionally, immune cell infiltration analysis revealed that the dysregulated immune cell proportions were identified, and macrophages could have a critical role in USCP pathogenesis.

**Supplementary Information:**

The online version contains supplementary material available at 10.1186/s40001-023-01043-4.

## Introduction

Uremia is one of the most challenging medical disorders and a growing public health issue worldwide [1]. It is often complicated by severe arteriosclerosis, consequently, cardiovascular and cerebrovascular complications are more likely to occur compared to the general population [2]. Arteriosclerosis refers to major pathological alterations in cardiovascular and cerebrovascular diseases with uremia, which cannot be fully explained by the classical risk factors, such as age, gender, smoking, obesity, dyslipidemia, hypertension, and diabetes [3]. A previous study described that during the development of atherosclerosis in patients with uremia, atherogenesis is stimulated by lipid disturbances, thrombogenesis, and synthesis of vasoactive substances, growth factors, and mediators of inflammation [4].

Atherosclerosis is a frequently encountered vascular condition that affects the intima of the arteries and can be life-threatening [5]. Its progression may lead to plaque destabilization, resulting in plaque rupture and thrombosis. Moreover, rupture of the carotid artery by an unstable plaque is one of the primary causes of stroke [6]. Even when the rupture of plaques does not result in a stroke, they likely contribute to plaque progression and arterial stenosis [7]. As is well established, uremic patients suffer from accelerated atherosclerosis; i.e., plaques form faster, and the disease is more severe [8]. Earlier studies have signaled that uremia plays a decisive role in stimulating plaque growth, intraplaque hemorrhage, and lesion instability [9]. Nevertheless, even when severe, unstable carotid plaque (USCP) patients are frequently asymptomatic, implying that they are unaware of the illness and do not seek medical help until alarming complications arise [10]. Therefore, there is an urgent need to distinguish stable carotid plaques (SCPs) from USCPs to prevent cerebrovascular diseases; indeed, early diagnosis and prompt treatment of USCPs are crucial. Accordingly, biomarkers are essential for early diagnosis and medical intervention for conditions such as uremia and USCP, which typically lacks overt clinical manifestations.

In recent years, microarray analysis has become a vital method for researching genetic modifications in various diseases owing to advancements in technologies [11]. Biomarkers for uremia have been identified in multiple studies. For instance, FZD10, FOXD4, PPP3R1, and UCP2 may be valuable uremia diagnostic biomarkers [12]. Another research reported that genes associated with USCP encompassed CD5L, S100A12, CKB, CEMIP, and SH3GLB1 [13]. However, no previous studies have identified the mechanism of uremia-induced USCP at the genetic level. An attempt was made to apply Limma analysis and the weighted gene co-expression network analysis (WGCNA) to screen the gene clusters of connected, shared, and correlating genes in uremia and USCP. Through this method, common risk genes associated with multiple disease phenotypes [14] have been identified.

To the best of our knowledge, this was the first study to simultaneously analyze uremia and USCP. More importantly, the identification of pivotal diagnostic biomarkers for uremia-related USCP was pursued through a series of bioinformatics and machine learning approaches.

## Methods

### Microarray data and data processing

**Fig. 1:**
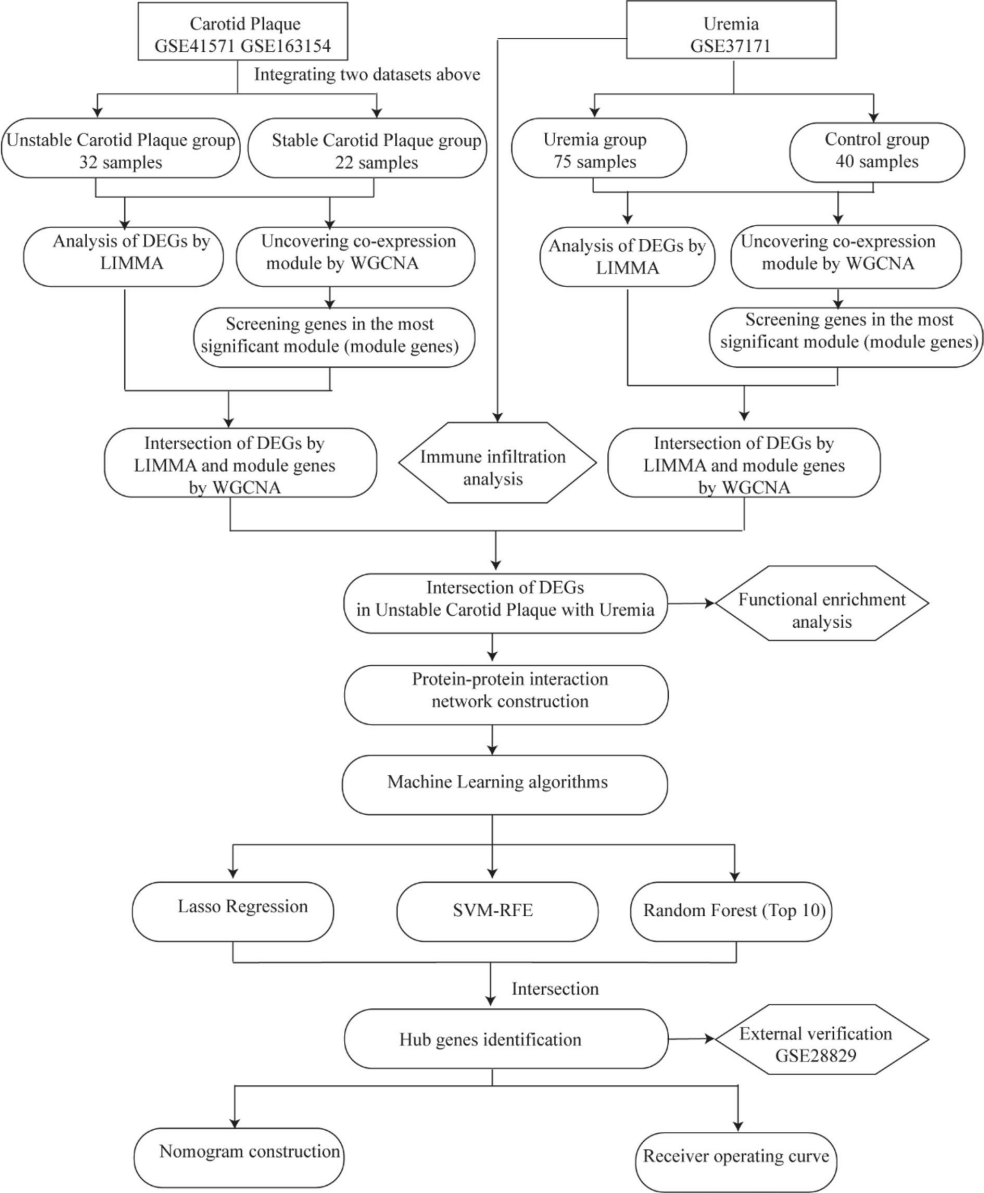
Study flowchart. *DEGs* differentially expressed genes, *LIMMA* linear models for microarray data, *WGCNA* weighted gene co-expression network analysis, *SVM*-*RFE* support vector machine-recursive feature elimination, *ROC* receiver operating characteristic curve

The study flowchart is illustrated in Fig. 1. Four microarray datasets (GSE37171, GSE41571, GSE163154, and GSE28829) were downloaded from the NCBI Gene Expression Omnibus (GEO; https://www.ncbi.nlm.nih.gov/geo/) database [15]. The search strategy was “Uremia” or “Carotid Plaque” AND “Homo sapiens” AND “Series” AND “Expression profiling by array”. Dataset GSE37171 [16] included data on 75 uremic patients and 40 controls. Datasets GSE41571 [17] and GSE163154 [18] included gene expression data on USCP and SCP patients. Data GSE28829 [19] was utilized as the external validation dataset. Detailed information on the datasets is presented in Table 1.

**Table 1:** Basic information on the GEO datasets used in the study

ID	GSE series	Disease	Samples	Source types	Platform	Group
1	GSE37171	Uremia	75 uremia patients and 40 normal controls	whole blood	GPL570	Discovery cohort
2	GSE41571	CP	5 USCP patients and 6 SCP patients	Plaque	GPL570	Discovery cohort
3	GSE163154	CP	27 USCP patients and 16 SCP patients	Plaque	GPL6104	Discovery cohort
4	GSE28829	CP	16 advanced carotid plaque patients and 13 early carotid plaque patients	Plaque	GPL570	Validation cohort

*CP* carotid plaque, *USCP* unstable carotid plaque, *SCP* stable carotid plaque

The data were preprocessed using “affy” in R (R Institute for Statistical Computing, Vienna, Austria) from the Bioconductor project to carry out background calibration and normalization. Multiple probes matching the same gene were calculated as a median expression. Gene symbols were converted from probes and then prepared into matrix files. After merging the GSE41571 and GSE163154 datasets, batch effects and other unwanted variations between the two datasets were eliminated using the R “surrogate variable analysis” (SVA) package from Bioconductor.

### Differentially expressed genes (DEGs) identification

The Bioconductor Limma package [20] was used to screen DEGs between uremia and the control in dataset GSE37171 and to identify DEGs between USAP and SAP in the merged dataset (GSE41571, GSE163154). The criteria for identification of DEGs were |Fold change|> 1.5 and P value < 0.05.

### Significant module identification via weighted gene co-expression network analysis (WGCNA)

WGCNA [21] is an efficient method for building co-expression networks extensively applied to analyze large datasets. Using WGCNA, respective significant module genes correlating with uremia and USCP were identified. First, each gene was analyzed by calculating the median absolute deviation (MAD) and eliminating 50% of those with the smallest MAD. The goodSamplesGenes function of WGCNA was then used to filter the DEG expression matrix to create a scale-free co-expression network. Next, a weighted adjacency matrix was built based on the soft-thresholding parameter β, originating from co-expression similarity, applying the pick-Soft-Threshold function. A topological overlap matrix (TOM) was constructed based on adjacency; TOM has the ability to measure the network connectivity of a gene defined as the sum of its adjacency with all other genes for network generation. Additionally, the corresponding dissimilarity (1-TOM) was determined. Then, hierarchical clustering and dynamic tree-cutting were used to identify the modules. Using the TOM-based dissimilarity measure, with 50 genes (Gene group) as the minimum size, average linkage hierarchical clustering was constructed for the Genes dendrogram; gene modules were formed by classifying genes with similar expression profiles. Finally, the eigengene network was visualized after merging some modules based on the dissimilarity of estimated module eigengenes.

### Gene Ontology (GO) and Kyoto Encyclopedia of Genes and Genomes (KEGG) pathway enrichment analysis

GO [22] contains structured and computable information about the functions of genes and their products. During GO analysis, biological process (BP), cellular component (CC), and molecular function (MF) are identified. As a knowledge base, KEGG [23] provides detailed information about pathways, as well as systematic analyses of gene functions. Functional enrichment analysis was performed with the R package ClusterProfiler (version 3.14.3) [24], and the top 10 GO terms were visualized using the R package “ggplot2” in each category. The selection criteria were: adjusted P value < 0.05 and false discovery rate < 0.25.

### Protein–protein interaction (PPI) network construction

PPI was constructed using the Search Tool for Retrieval of Interacting Genes (String) database [25] (version 11.5; www.string-db.org), an online tool for identifying protein interactions; 0.40 was set as the minimum required interaction score. Genes that did not interact with each other were hidden. Next, the file of interaction data was downloaded and visualized using Cytoscape [26] (http://www.cytoscape.org). The top 30 genes were identified using three algorithms (betweenness, closeness, and degree) in CytoHubba [27], a plug-in of Cytoscape. Finally, the DEGs were visualized by intersecting the three algorithms using a Venn diagram.

### Machine learning

For the identification of candidate hub genes, three different machine learning algorithms were utilized to minimize the risk of bias in potential diagnostic genes. The Support Vector Machine-Recursive Feature Elimination (SVM-RFE) [28] algorithm of the support vector machine was used to identify the optimal variables by deleting SVM-generated eigenvectors. The least absolute shrinkage and selection operator (Lasso) [29] regression analysis was performed using the “glmnet” R package; regularization was utilized to reduce prediction errors. With random forest [30], high dimensions of data can be handled, predictive models can be developed, and the importance of each variable can be predicted. The genes from all three algorithms were eventually intersected for further analysis.

### Receiver operating characteristic curve (ROC) evaluation and nomogram construction

The Student’s *t*-test was used to compare the expression level of each candidate diagnostic gene between the USCP and the SCP groups. In order to evaluate the diagnostic value of each candidate gene, a ROC curve was established; the area under the curve (AUC) and the 95% confidence interval (CI) were subsequently calculated. Using the R package “rms”, a nomogram was constructed to provide a clinical perspective on the diagnosis of uremia with USAP. Each gene was scored based on its expression, and after the accumulation of all genes, the total score could be used to predict the incidence of uremia with USAP. Finally, a ROC curve was constructed for the nomogram. Lastly, the validation dataset GSE28829 was applied to analyze the diagnostic value of each candidate gene via the ROC curve and nomogram.

### Immune infiltration analysis

Using the “Cibersort” algorithm, the normalized gene expression matrix was converted into the composition of infiltrating immune cells [31]. With the R package “Cibersort”, the percentage of different types of immune cells was calculated and displayed in a bar plot between USCP and SCP. Next, the “vioplot” R package was adopted to compare and visualize the proportion of immune cells between USCP and SCP. Then, using the R package “corrplot” [32], a heatmap of the correlation between immune cells and USCP pathogenesis was constructed. Finally, the correlation between immune cell infiltrations and the hub diagnostic genes was analyzed.

## Results

### DEGs identification via Limma in uremia and USCP

**Fig. 2:**
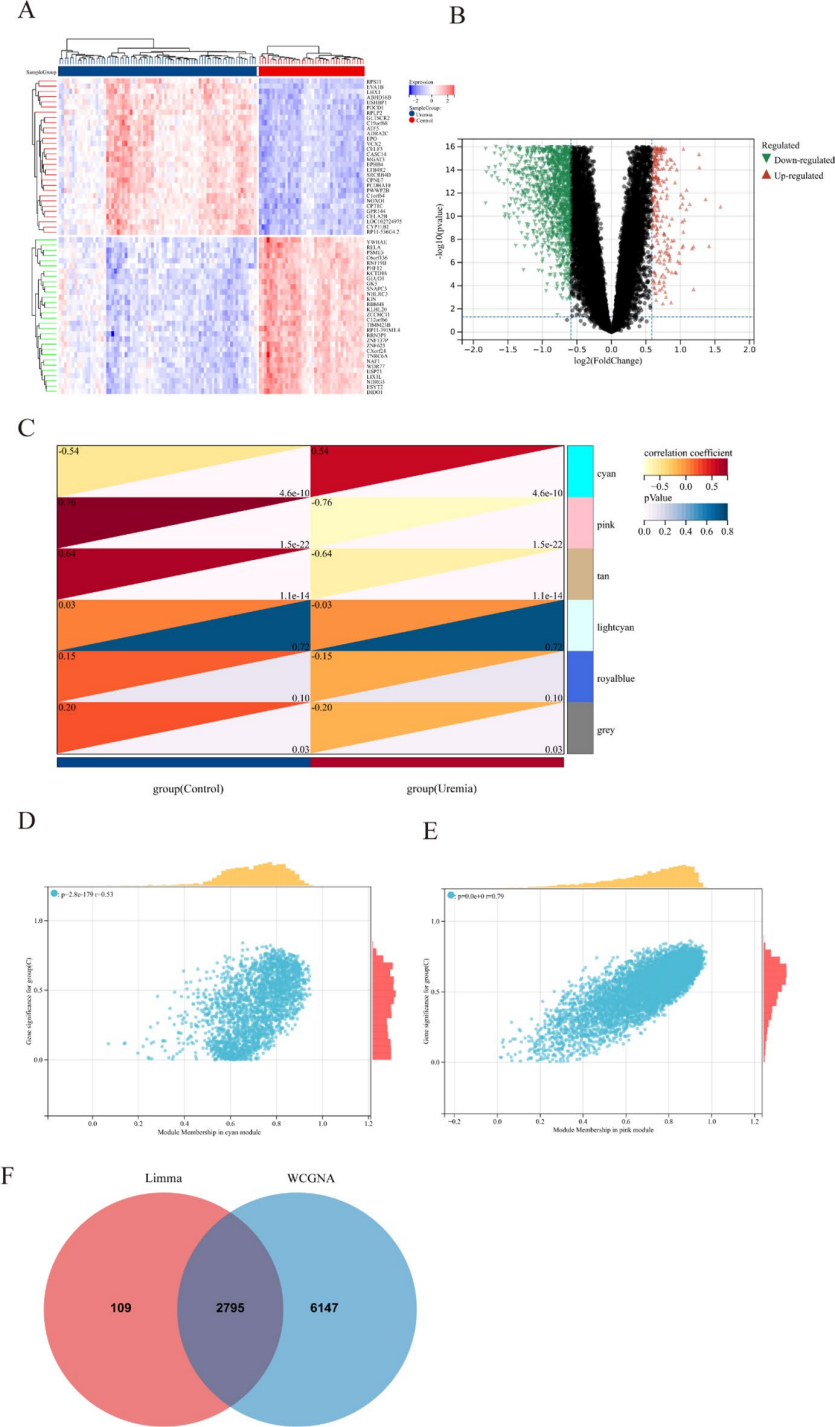
The heatmap and volcano plot of DEGs between uremia and the control, module genes recognition in uremia via WGCNA. **A** Heatmap showing the top 30 up-regulated and down-regulated DEGs in uremia compared with the control. Red blocks indicate up-regulation between the two groups, while blue blocks indicate down-regulation. **B** The volcano plot displaying all DEGs between uremia and the control. Significantly up-regulated and down-regulated DEGs with significant upregulations or downregulations are marked in red and green, respectively. **C** The heatmap of uremia-associated modules. Numbers in the top left corner indicate module–uremia correlation, and the P value in the lower right corner indicates the correlation. In uremia, cyan and pink modules show the most significant correlation. **D**–**E** The correlation between module membership in the cyan/pink module and gene significance in uremia. **F** The Venn plot displays that showing the 2795 DEGs were identified from the intersection between DEGs via Limma and significant module genes via WGCNA in uremia. *DEGs* differentially expressed genes, *WGCNA* weighted gene co-expression network analysis

**Fig. 3:**
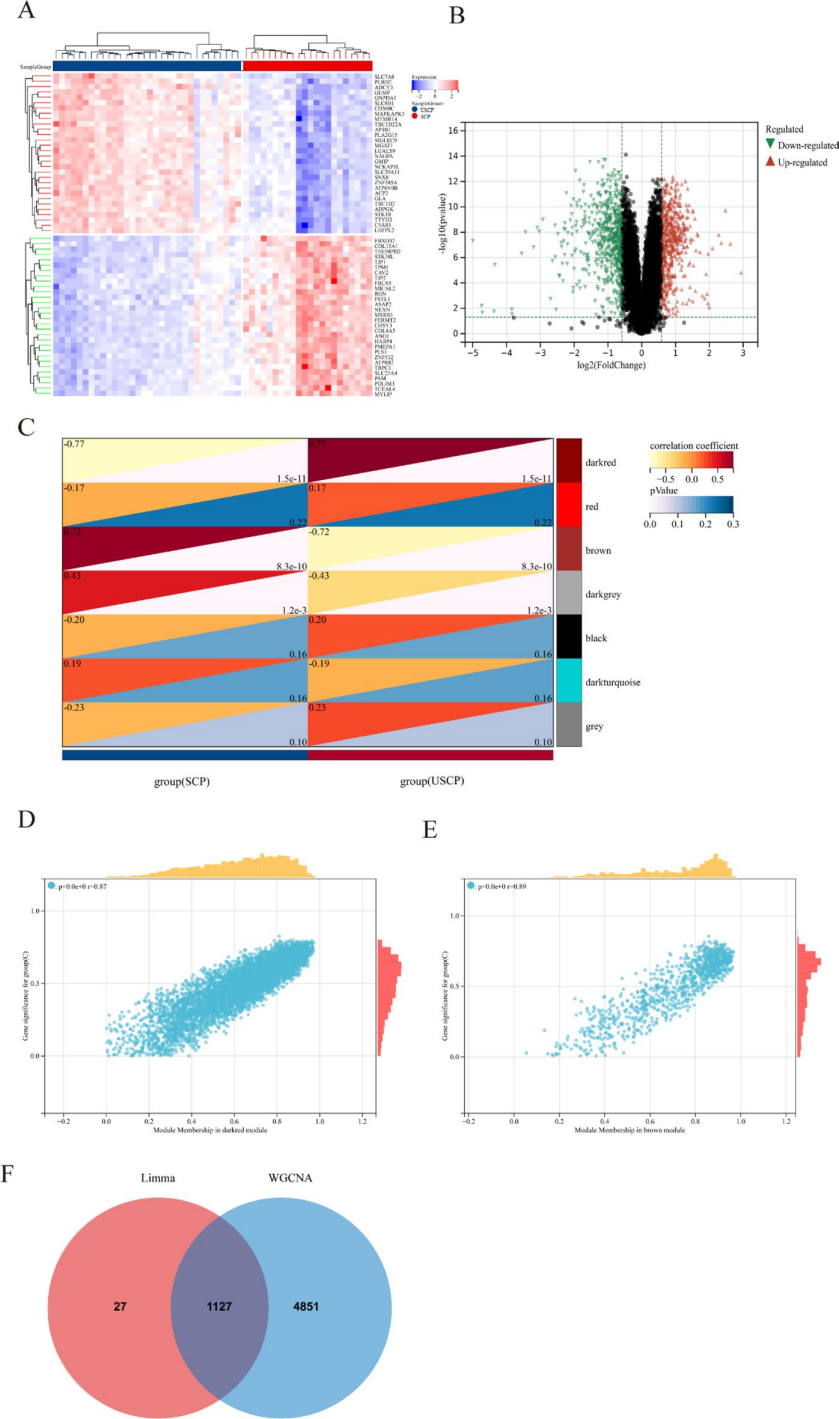
The heatmap and volcano plot of DEGs between USCP and SCP, module genes recognition in uremia via WGCNA. **A** Heatmap showing the top 30 up-regulated and down-regulated DEGs in USCP compared with SCP. Red blocks indicate up-regulation between the two groups, while blue blocks indicate down-regulation. **B** The volcano plot displaying all DEGs between USCP and SCP. Significantly up-regulated and down-regulated DEGs with significant upregulations or downregulations are marked in red and green, respectively. **C** The heatmap of USCP-associated modules. Numbers in the top left corner indicate module–USCP correlation, and the P value in the lower right corner indicates the correlation. In USCP, dark red and brown modules show the most significant correlation. **D**–**E** The correlation between module membership in dark red/brown module and gene significance in USCP. **F** The Venn plot illustrating the 1127 DEGs were identified from the intersection between DEGs via Limma and significant module genes via WGCNA in USCP. *USCP* unstable carotid plaque, *SCP* stable carotid plaque, *DEGs* differentially expressed genes, *WGCNA* weighted gene co-expression network analysis

2904 DEGs were screened between the uremia and control groups, of which 495 were up-regulated, and 2409 were down-regulated. A total of 1154 DEGs were screened between the USCP and SCP groups, of which 544 were up-regulated, and 610 were down-regulated. A heatmap and volcano plot were used to display the top 30 up-regulated or down-regulated DEGs and all DEGs, respectively (uremia: Fig. 2A, B, USCP: Fig. 3A, B).

### Identification of significant module genes in uremia and USCP via WGCNA

Significant module genes of uremia and USCP were identified using WGCNA. The gray module failed to cluster genes regarded as a junk module. Meanwhile, the cyan module exhibited the most significant positive correlation with uremia (*r* = 0.54, *p* = 4.6 × 10–10), while negative correlation was most prominent in the pink module (*r* = −0.76, *p* = 1.5 × 10–22) (Fig. 2C). Figure 2D, E depicts the correlation between module membership in gene significance and the cyan/pink module in uremia. Meanwhile, the dark red module exhibited the most significant positive correlation with USCP (r = 0.77, p = 1.5 × 10–11), whereas negative correlation was most prominent in the brown module (*r* = −0.72, *p* = 8.3 × 10–10) (Fig. 3C). Figure 3D, E delineates the correlation between module membership in gene significance and the cyan/pink module in USCP. As a result, 8942 uremia-related genes and 5978 USCP-related genes were selected.

Finally, the intersection of 2904 DEGs and 8942 module genes regarding uremia yielded 2795 uremia-related DEGs (Fig. 2F), and the intersection of 1154 DEGs and 5978 module genes regarding USCP yielded 1127 USCP-related DEGs (Fig. 3F). The soft threshold selection and gene cluster tree are displayed in Additional file 1: Figures S1 and S2.

### Functional enrichment analysis of uremia-related DEGs in USCP

**Fig. 4:**
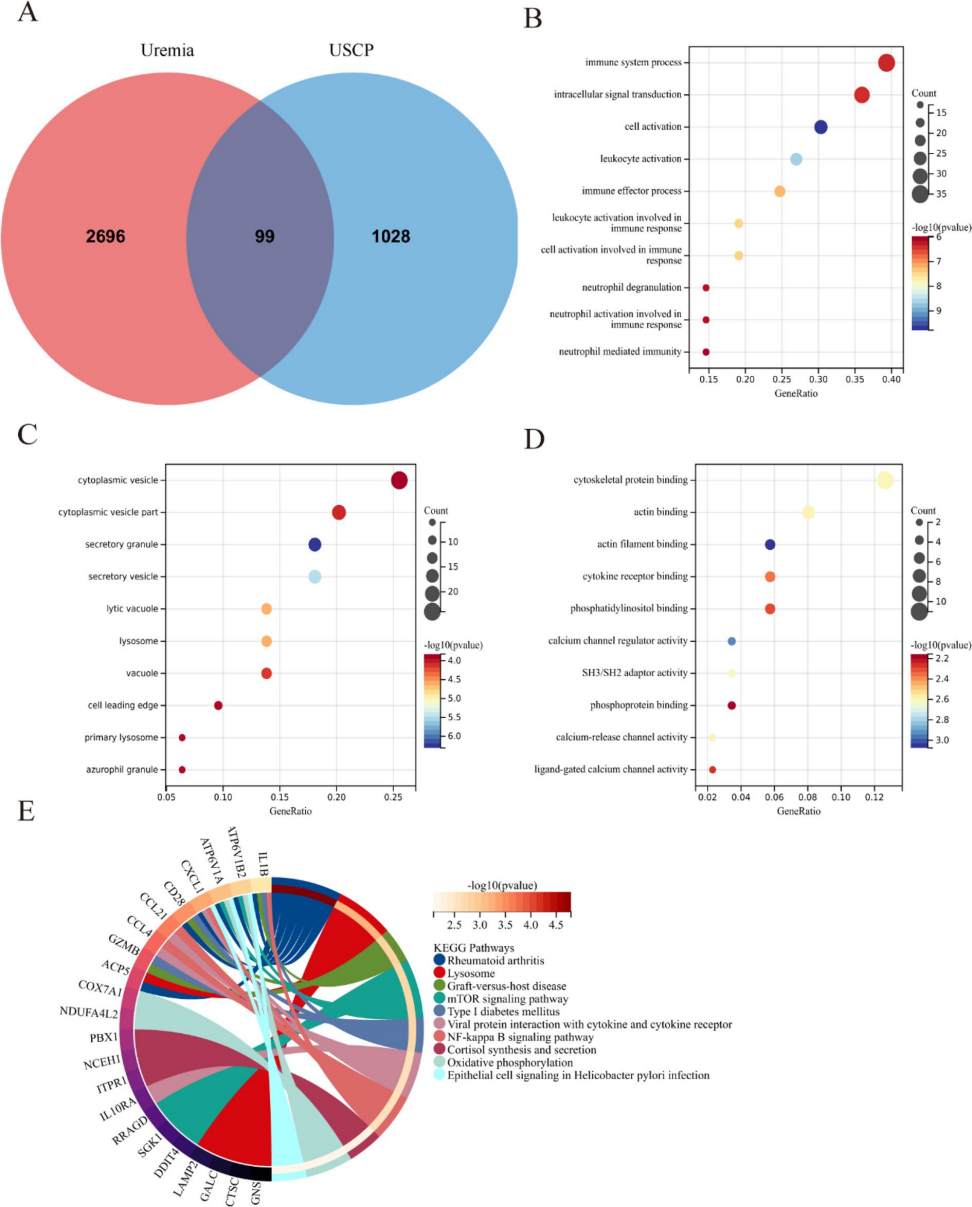
Functional enrichment analysis of uremia-related DEGs in USCP. **A** The Venn plot displaying the intersection of DEGs between uremia and USCP yielded 99 uremia-related DEGs in USCP. **B**–**D** GO analysis (BP, CC, and MF) of uremia-related DEGs in USCP. The X-axis represents the gene ratio,; Y-axis refers to different ontologies,; the circle size represents the gene number, and; the color indicates the significance. **E** KEGG pathway analysis of uremia-related DEGs in USCP. *USCP* unstable carotid plaque, *DEGs* differentially expressed genes, *BP* biological function, *CC* cellular component, *MF* molecular function, *GO* Gene Ontology, *KEGG* Kyoto Encyclopedia of Genes and Genomes

The intersection of 2795 uremia-related DEGs and 1127 USCP-related DEGs yielded 99 uremia-related DEGs in USCP (Fig. 4A). Significantly enriched GO terms of the 99 uremia-related DEGs for BP included “immune system process”, “intracellular signal transduction”, and “cell activation”. Moreover, “cytoplasmic vesicle”, “cytoplasmic vesicle part”, and “secretory vesicle” were enriched in CC, while the MF of DEGs were highly associated with “cytoskeletal protein binding”, “actin binding”, and “actin filament binding” (Fig. 4B–D, Additional file 1: Table S1). Figure 4E displays the functional pathway analysis of the 99 DEGs, mainly enriched in “Rheumatoid arthritis”, “Lysosome”, and “Graft-versus-host disease”.

### PPI network construction and hub gene selection

**Fig. 5:**
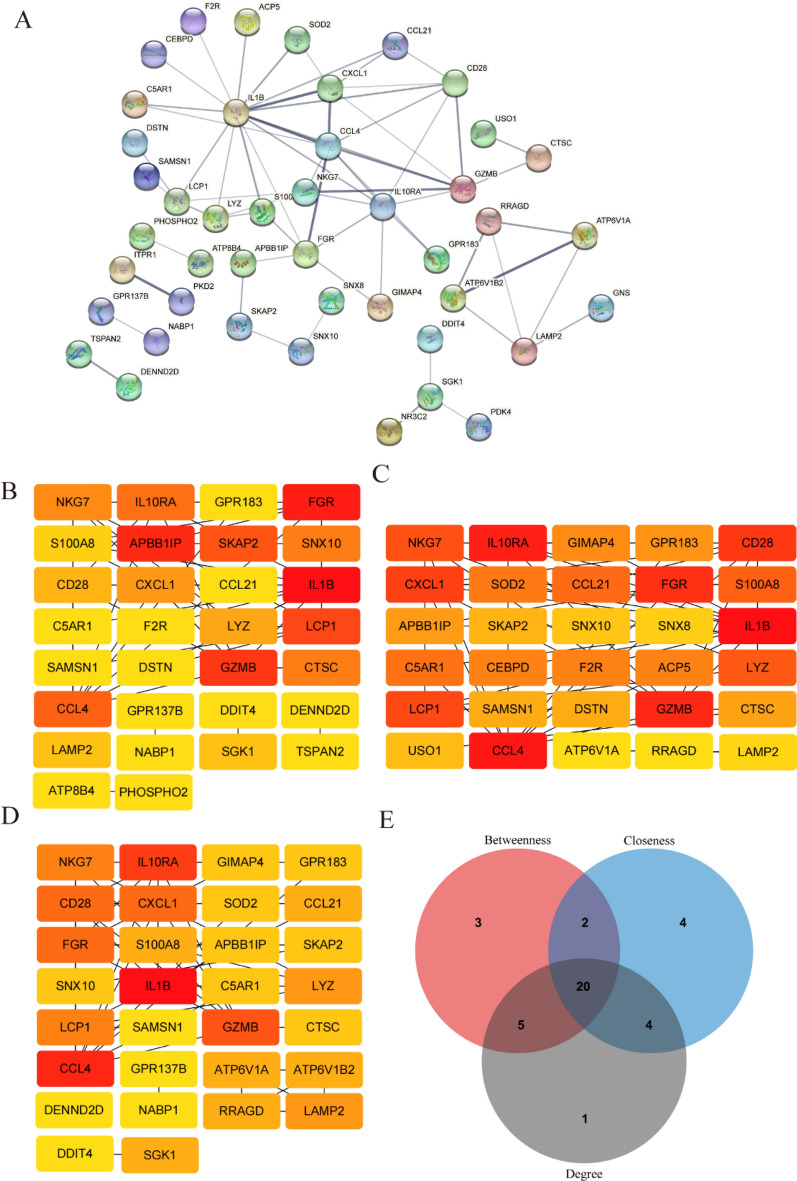
PPI network construction and hub gene selection. **A** The whole PPI network of 44 uremia-related DEGs in USCP was visualized via String. The remaining 55 DEGs were eliminated due to a lack of interaction. **B**–**D** With the CytoHubba plug-in of Cytoscape, we selected the top 30 DEGs were selected using three different algorithms. Panels **B–D** illustrate betweenness, closeness, and degree algorithms. In the algorithm, darker colors indicate more significant weights. **E** Based on the intersection of genes from the three algorithms, 20 DEGs were chosen for further analysis. *PPI* protein–protein interaction network, *DEGs* differentially expressed genes, *USCP* unstable carotid plaque

Based on the 99 DEGs, a PPI network was preliminarily constructed to identify hub DEGs for uremia with USCP. 44 DEGs were screened after interacting with the others (Fig. 5A), and 45 DEGs were eliminated due to lack of interaction. Moreover, three different algorithms (degree, betweenness, closeness) were used in the CytoHubba plug-in to identify intersecting DEGs. The top 30 node genes from the betweenness, closeness and degree algorithms are visualized and presented in Fig. 5B–D. The intersection of 30 genes from the three algorithms was depicted using a Venn diagram, and 20 genes were selected as candidate hub genes (Fig. 5E). Detailed information on the 20 genes is listed in Additional file 1: Table S2.

### Candidate hub genes selection via machine learning

**Fig. 6:**
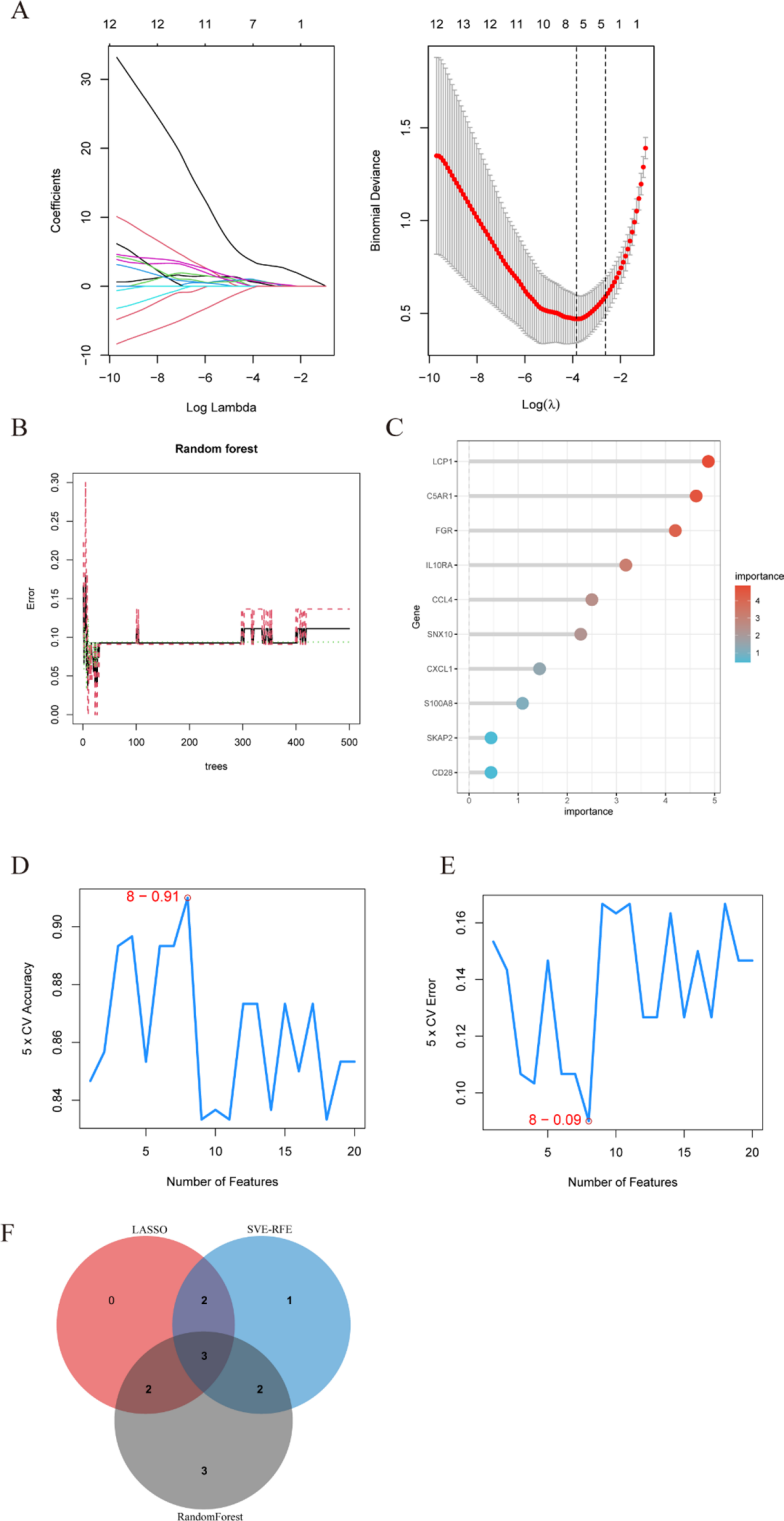
Candidate diagnostic biomarker identification via machine learning algorithms. **A** Based on the Lasso regression algorithm, 7 genes corresponding to the lowest point of the curve were identified as the most accurate biomarkers for USCP with the diagnosis of uremia with USCP. **B**, **C** The random forest algorithm shows the error in USCP. Based on the random forest algorithm's importance score, we selected and ranked the top ten genes. **D**, **E** 8 genes were selected based on SVM-RFE with the lowest error and highest accuracy. **F** Based on the intersection of genes from three algorithms, three hub genes (FGR, LCP1, and C5AR1) were selected for the next step of nomogram construction and diagnostic value evaluation. *SVM*-*RFE* support vector machine-recursive feature elimination, *FGR* feline Gardner-Rasheed, *LCP1* lymphocyte cytosolic protein 1, *C5AR1* complement C5a receptor 1

Lasso regression, random forest, and SVM-RFE algorithms were adopted to screen candidate hub genes for ROC evaluation. According to the Lasso regression algorithm, 7 potential hub genes corresponding to the lowest point of the curve wereidentified as the most suitable biomarkers for USCP with uremia diagnosis. Figure 6 A illustrates the Lasso regression results. DEG gene importance was calculated using random forest.

The random forest algorithm revealed the error in USCP (Fig. 6B). A list of the ten most important DEGs is shown in Fig. 6C. As a result of SVM-RFE, the top 8 DEGs had the lowest error and highest accuracy in diagnosing USCP with uremia (Fig. 6D, E). Finally, based on the intersection of genes from the three algorithms, three hub genes (FGR, LCP1, and C5AR1) were selected for nomogram construction and diagnostic value evaluation (Fig. 6F).

### The diagnostic value evaluation and nomogram construction

**Fig. 7:**
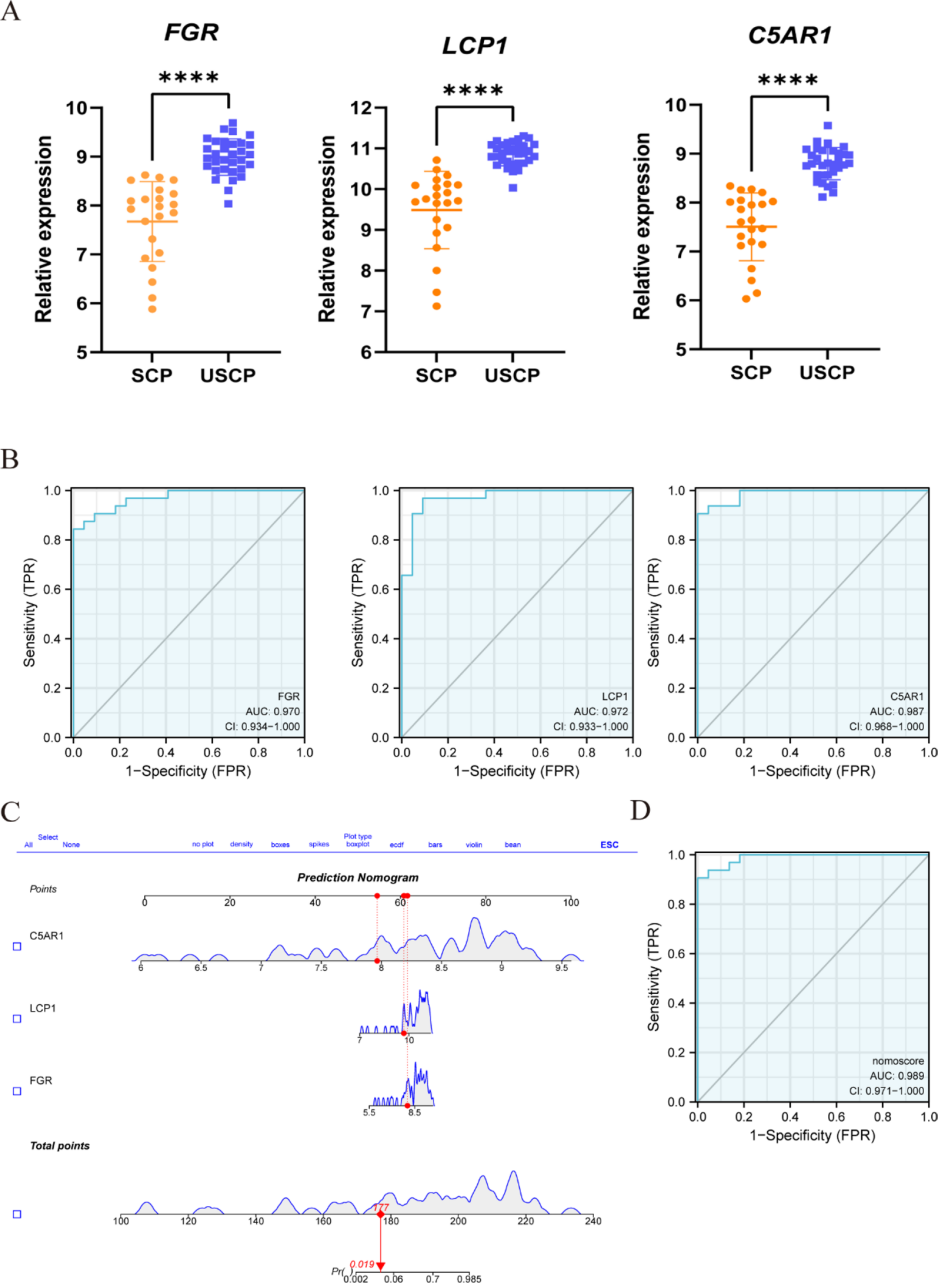
The diagnostic value of candidate biomarkers and the nomogram construction. **A** Differences in expression between USCP and SCP for the three genes. ****, P < 0.0001. **B** The diagnostic value of the three genes in USCP with uremia from the ROC curve. Each panel displays the AUC under the curve and 95% CI. **C** Based on the three genes, a nomogram was constructed for diagnosing USCP with uremia. **D** The diagnostic value of the nomogram in USCP with uremia from the ROC curve. *SCP* stable carotid plaque, *USCP* unstable carotid plaque, ROC receiver operating characteristic curve, *AUC* area under the curve, *CI* confidence interval

**Fig. 8:**
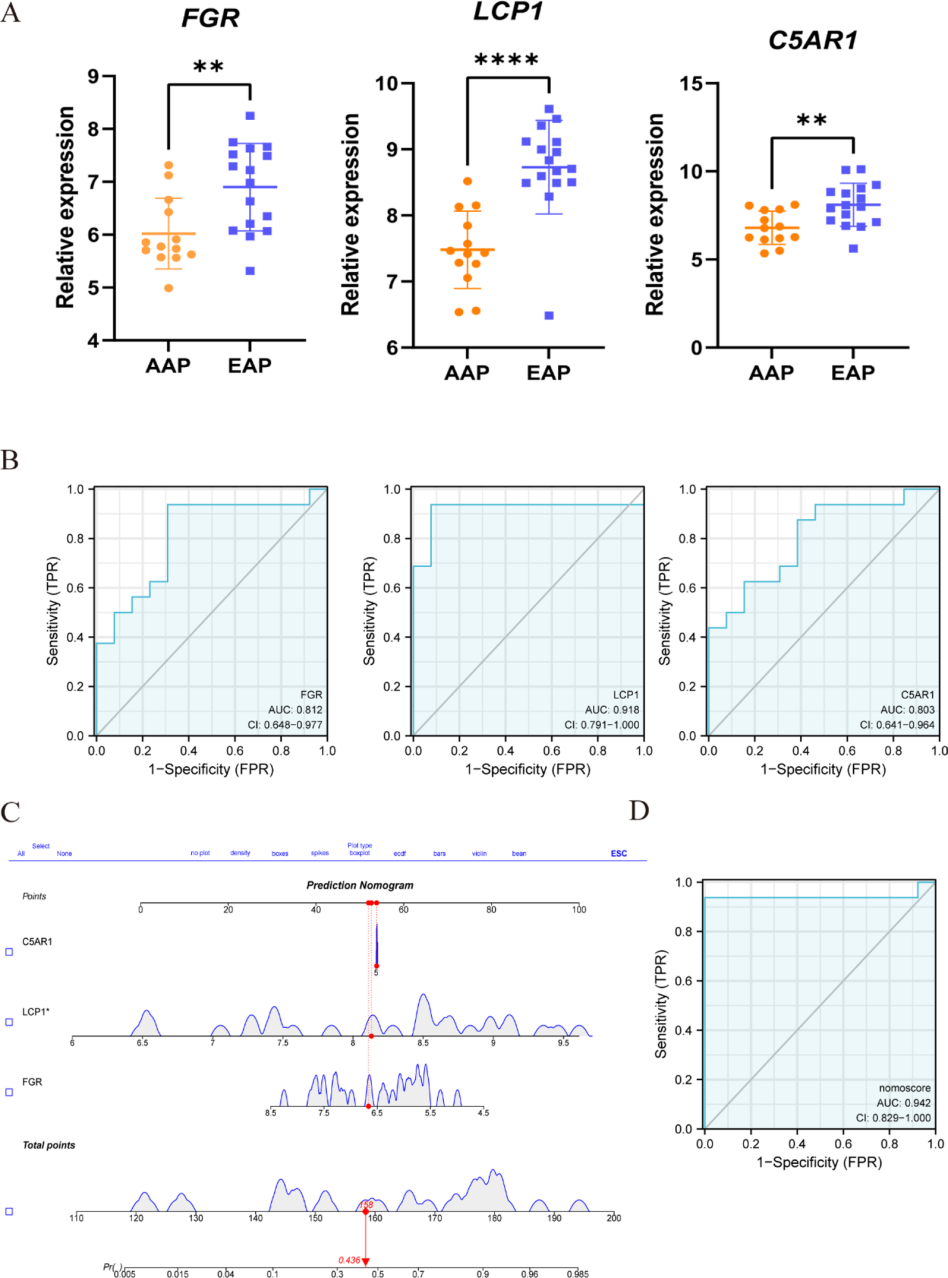
The diagnostic value of candidate biomarkers and the nomogram construction in the external validation dataset GSE28829. **A** Differences in expression between EAP and AAP for the three genes. **, P < 0.01; ****, P < 0.0001. **B** The diagnostic value of the three genes in AAP from the ROC curve. Each panel displays the AUC under the curve and 95% CI. **C** Based on the three genes, a nomogram was constructed for AAP. **D** The ROC curve of the nomogram in AAP. *AAP* advanced-stage atherosclerotic plaque, EAP early-stage atherosclerotic plaques, *ROC* receiver operating characteristic curve, *AUC* area under the curve, *CI* confidence interval

Three hub genes were up-regulated in USCP compared to SCP (Fig. 7A). The diagnostic value of each candidate hub gene was assessed using ROC curves. Figure 7B shows the AUC and 95% CI for each gene: FGR (AUC: 0.970, 95% CI 0.934–1.000); LCP1 (AUC: 0.972, 95% CI 0.933–1.000); C5AR1 (AUC: 0.987, 95% CI 0.968–1.000). Each gene presented a satisfactory diagnostic performance based on ROC curve analysis. Finally, the nomogram was built (Fig. 7C), and the AUC under the nomogram was 0.989 (95% CI 0.971–1.000), yielding a high clinical diagnostic value (Fig. 7D). As part of the validation process, the validation dataset of early-stage and advanced-stage atherosclerotic plaques (GSE28829) was adopted to evaluate the diagnostic value via ROC curve analysis. ROC analysis of the nomogram yielded an AUC of 0.942, demonstrating an equally high clinical diagnostic value (Fig. 8).

### Immune cell infiltration analysis

**Fig. 9:**
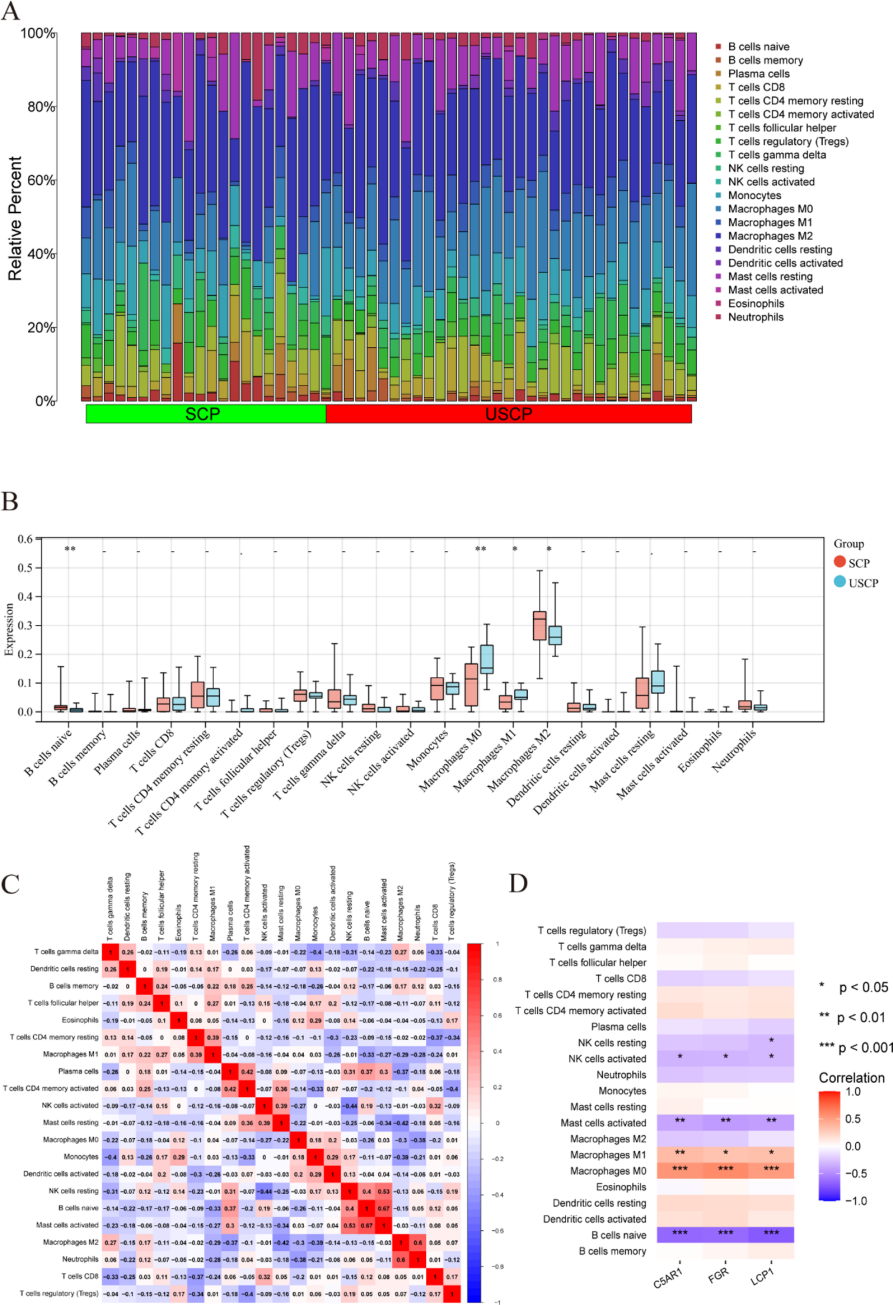
Alterations in the immunological features from SCP to USCP and correlations between the hub DGEs and immunological features in USCP. **A** The bar plot showing the proportion of immune cells in different samples. **B** The boxplot comparing the expression of immune cells between USCP and SCP. *P < 0.05; **P < 0.01. **C** The correlated heatmap represents the correlation between different immune cells in USCP pathogenesis. **D** Correlation analysis of immune cell infiltrations with the three hub DEGs. *P < 0.05; **P < 0.01; ***P < 0.001. *SCP* stable carotid plaque, *USCP* unstable carotid plaque, *DEGs* differentially expressed genes

A bar plot (Fig. 9A) was used to display the percentage of 21 types of immune cells in each sample following the application of the Cibersort algorithm; in all samples, CD4 naive T cells were absent because their proportion was zero. The boxplot showed that the proportion of M0 and M1 macrophages were higher in USCP compared with the control, while the proportion of naive B cells and M2 macrophages was lower in SCP (Fig. 9B). Correlation analysis showed that activated mast cells had the highest positive correlation with naive B cells (*r* = 0.67), while resting NK cells had the highest negative correlation with activated NK cells (*r* = −0.44) (Fig. 9C). In summary, focusing on macrophage regulation could serve as a potential approach for USCP treatment. Additionally, immune cell infiltration was positively correlated with all three hub DEGs (Fig. 9D).

## Discussion

Uremia is frequently complicated by severe arteriosclerosis [33, 34]. Dyslipidemia, inflammation, and oxidative stress have been reported to be the risk factors for arteriosclerosis development in uremic patients [35]. Patients with uremia suffer from accelerated atherosclerosis; atherosclerosis progression may trigger the rupture of plaques, the formation of thrombi in the lumen of vessels, and the occurrence of clinical events, which significantly contribute to the morbidity and mortality of patients [36]. To the best of our knowledge, this is the first study to apply bioinformatics analysis methods and machine learning algorithms to uremia and USCP. Moreover, all the dataset samples used in this study were peripheral blood samples. Accordingly, only the analysis of the expression of the hub genes in peripheral blood from uremic patients was required to estimate the probability of USCP incidence in uremic patients, which is an effective and practical clinical approach. The total score could be determined by adding the scores of each gene after establishing a nomogram. Uremic patients with high total scores should be screened, and the prognosis for this patient population can be enhanced with early treatment. Therefore, the nomogram could be extensively employed in clinical practice.

Using multiple integrated bioinformatics and machine learning methods, the current study discovered three biomarkers (FGR, LCP1, and C5AR1). A nomogram was constructed, and its diagnostic value for USCP in uremic patients was evaluated. Additionally, external validation was performed using an additional dataset (GSE28829), which uncovered close relationships between the three hub genes and atherosclerosis progression.

FGR is a member of the protein tyrosine kinases (PTKs) family. FGR gene expression has been shown to be restricted to peripheral blood granulocytes and monocytes, and tissue macrophages [37]. It further contributes to the regulation of immune responses, including neutrophils, monocytes, macrophages, mast cells, phagocytosis, cell adhesion, and migration [38]. Crainciuc et al. [39] revealed that inflammatory injury was prevented in mice through interference with FGR. Meanwhile, Medina et al. [40] found that FGR deficiency in hematopoiesis impeded atherosclerosis plaque formation by limiting endothelial adhesion and transmigration. In the present study, FGR was up-regulated in USCP patients, implying that inflammatory injury and dysregulated immunity resulted in USCP.

LCP1 encodes the actin-binding protein. Although LCP1 is widely expressed across all hematopoietic lineages, numerous tumor cells are highly up-regulated by LCP1. Interestingly, redox-modified LCP1 inhibits tumor cell actin-based functions [41]. Ge et al. [42] discovered that LCP1 promotes osteosarcoma progression via the activation of the JAK2/STAT3 signaling pathway. Zeng et al. [43] reported that several immune marker sets were highly correlated with LCP1, which was associated with tumor-infiltrating lymphocytes in gastric cancer. In macrophages, LCP1 is required for podosome formation and function [44]. Transcriptome analysis of human aorta atherosclerotic lesions indicated that the candidate gene LCP1 participates in phenotypic changes of smooth muscle cells, immune and inflammatory reactions, as well as oxidative processes [45]. In the present study, LCP1 was up-regulated in USCP patients. However, the relationship between LCP1 and USCP is still unknown.

C5AR1, a G protein-coupled receptor for complement C5a, is a potent immune mediator [46]. A crucial component of innate and adaptive immunity is the complement system, which identifies and eliminates pathogens. C5a expression was significantly elevated in patients with severe atherosclerosis, and C5a was an independent predictor of cardiovascular events [47]. Recruitment of circulating monocytes into the artery wall is a key link in the formation of atherosclerosis [48]. C5a is notably a potent neutrophil chemotactic factor; increased C5a receptor expression facilitates the entry of monocytes into the arterial intima [49], which may generate the conditions for atherosclerosis formation. Interestingly, C5a is positively correlated with atherosclerotic plaque instability [50]. The findings of the current study suggested that C5AR1 expression was higher in USCP than in SCP, indicating that the innate and adaptive immune responses were activated, resulting in USCP.

Chronic systemic inflammation and immune activation are hallmarks of uremia [51]. During the development and progression of atherosclerosis, inflammation and immune responses also play a significant role [52]. As is well documented, immune-related inflammatory state is a key mechanism for the occurrence and development of arteriosclerosis in uremic patients [53]. GO analysis indicated that DEGs were mainly related to immune regulation, such as immune system process, intracellular signal transduction, and cell activation. This study also analyzed the immune infiltration of USCP. The results demonstrated that M0 and M1 macrophages were higher in USCP compared with the control, while the proportion of M2 macrophages was lower in SCP. The proportion of immune cells contributes critically to the initiation and progression of USCP [54]. A previous study has established that the proportion of M0 and M1 macrophages is reportedly higher in USCP, while the proportion of M2 macrophages is lower [55], which is consistent with the findings of the current study. M1 macrophages may play a significant role in the initiation and progression of atherosclerosis, while M2 macrophages are hypothesized to stabilize plaques by virtue of their anti-inflammatory effect [56]. In short, focusing on macrophage regulation could provide potential approaches for the treatment of USCP.

The interrelationship between the three identified hub genes and immunity was examined. The three hub genes (FGR, LCP1, and C5AR1) were positively correlated with immune-related M0 and M1 macrophages. Numerous studies have signaled that plaque stability can be increased by inhibiting M1 macrophage polarization [57, 58]. Therefore, immune-related genes are speculated to be highly involved in the progression of USCP. Most importantly, they can assist in the diagnosis of USCP with uremia and the development of targeted therapies.

However, there were several limitations in this study that need to be taken into account. To begin, the sample size for the unstable and stable carotid plaques was not large; only two datasets were available. Secondly, the basic clinical information of samples in different groups was limited which may cause population bias. Thirdly, although the validation dataset (GSE28829) was used to assess the diagnostic value, the diagnostic biomarkers and underlying mechanisms should be further validated through experimental studies.

## Conclusion

Our study systematically identified three candidate hub genes (FGR, LCP1, and C5AR1) and established a nomogram to assist in the diagnosis of USCP with uremia using various bioinformatic analyses and machine learning algorithms. The findings herein provide a theoretical basis for future studies on potential diagnostic candidate genes for USCP in uremic patients. Additionally, immune cell infiltration analysis revealed the dysregulated immune cell proportions and that macrophages may be implicated in the pathogenesis of USCP.

## Supplementary Information

**Additional file 1: Table S1.** Functional enrichment analysis of Uremia-related DEGs in USCP. **Table S2.** Complete list of DEGs from three algorithms via CytoHubba plug-in. **Figure S1.** Soft threshold selection and gene cluster tree via WGCNA of Uremia. **Figure S2.** Soft threshold selection and gene cluster tree via WGCNA of USCP.

## Data Availability

Datasets used in the study (GSE37171, GSE41571, GSE163154, and GSE28829) can be downloaded without restriction from the public GEO database. Upon reasonable request, data processing procedures are available from the first and corresponding authors.
