# Supplementary material for: Immune-associated biomarkers identification for diagnosing carotid plaque progression with uremia through systematical bioinformatics and machine learning analysis
Source: Eur J Med Res. 2023 Feb 23;28:92. doi: 10.1186/s40001-023-01043-4 (PMC9948329; doi:10.1186/s40001-023-01043-4)
Supplement: Supplementary file 1 — Additional file 1: Table S1. Functional enrichment analysis of Uremia-related DEGs in USCP. Table S2. Complete list of DEGs from three algorithms via CytoHubba plug-in. Figure S1. Soft threshold selection and gene cluster tree via WGCNA of Uremia. Figure S2. Soft threshold selection and gene cluster tree via WGCNA of USCP. [file 40001_2023_1043_MOESM1_ESM.docx]

**Additional Material**

**Additional Table S1.** Functional enrichment analysis of Uremia-related DEGs in USCP.

| Description | Count | Gene |  |
| --- | --- | --- | --- |
| **Biological Process** |  |  |  |
| cell activation | 27 | SH2B3/ITPR1/PURA/CD28/APBB1IP/DOCK8/GNS/F2R/ATP8B4/FGR/CXCL1/CTSC/CCL21/S100A8/IQGAP2/LCP1/IGFBP2/IL1B/SKAP2/LAMP2/SAMSN1/C5AR1/GPR183/SDCBP/LYZ/PNP/VNN1 |  |
| leukocyte activation | 24 | PURA/CD28/APBB1IP/DOCK8/GNS/ATP8B4/FGR/CXCL1/CTSC/CCL21/S100A8/IQGAP2/LCP1/IGFBP2/IL1B/SKAP2/LAMP2/SAMSN1/C5AR1/GPR183/SDCBP/LYZ/PNP/VNN1 |  |
| leukocyte activation involved in immune response | 17 | CD28/APBB1IP/GNS/ATP8B4/FGR/CXCL1/CTSC/S100A8/IQGAP2/LCP1/LAMP2/C5AR1/GPR183/SDCBP/LYZ/PNP/VNN1 |  |
| cell activation involved in immune response | 17 | CD28/APBB1IP/GNS/ATP8B4/FGR/CXCL1/CTSC/S100A8/IQGAP2/LCP1/LAMP2/C5AR1/GPR183/SDCBP/LYZ/PNP/VNN1 |  |
| immune effector process | 22 | CD28/APBB1IP/GNS/DDIT4/ATP8B4/FGR/CXCL1/CTSC/CYFIP2/S100A8/IQGAP2/LCP1/IL1B/LAMP2/GZMB/IFI27/C5AR1/GPR183/SDCBP/LYZ/PNP/VNN1 |  |
| intracellular signal transduction | 32 | SH2B3/ITPR1/LBH/ARHGEF37/CD28/DOCK8/PLAGL1/PKD2/F2R/CCL4/DDIT4/IL10RA/ARHGEF3/SOD2/KANK2/FGR/CXCL1/CCL21/NR3C2/S100A8/USO1/LCP1/IL1B/CYP1B1/RRAGD/STK38L/C5AR1/RNF149/GPR183/SDCBP/CTNNAL1/VNN1 |  |
| immune system process | 35 | SH2B3/PDGFD/PURA/CD28/APBB1IP/DOCK8/GNS/CCL4/DDIT4/ATP8B4/FGR/CXCL1/CTSC/CCL21/CYFIP2/CEBPD/S100A8/IQGAP2/LCP1/IGFBP2/IL1B/SNX10/SKAP2/LAMP2/GZMB/SAMSN1/IFI27/PBX1/C5AR1/TSPAN2/GPR183/SDCBP/LYZ/PNP/VNN1 |  |
| neutrophil degranulation | 13 | GNS/ATP8B4/FGR/CXCL1/CTSC/S100A8/IQGAP2/LAMP2/C5AR1/SDCBP/LYZ/PNP/VNN1 |  |
| neutrophil activation involved in immune response | 13 | GNS/ATP8B4/FGR/CXCL1/CTSC/S100A8/IQGAP2/LAMP2/C5AR1/SDCBP/LYZ/PNP/VNN1 |  |
| neutrophil mediated immunity | 13 | GNS/ATP8B4/FGR/CXCL1/CTSC/S100A8/IQGAP2/LAMP2/C5AR1/SDCBP/LYZ/PNP/VNN1 |  |
| **Cellular Component** |  |  | |
| secretory granule | 17 | ITPR1/GNS/ATP8B4/FGR/CXCL1/CTSC/S100A8/IQGAP2/SNX10/PAM/LAMP2/GZMB/C5AR1/SDCBP/LYZ/PNP/VNN1 | |
| secretory vesicle | 17 | ITPR1/GNS/ATP8B4/FGR/CXCL1/CTSC/S100A8/IQGAP2/SNX10/PAM/LAMP2/GZMB/C5AR1/SDCBP/LYZ/PNP/VNN1 | |
| lytic vacuole | 13 | GNS/GPR137B/CTSC/ATP6V1A/GALC/ATP6V1B2/IL1B/RRAGD/LAMP2/ACP5/SDCBP/LYZ/VNN1 | |
| lysosome | 13 | GNS/GPR137B/CTSC/ATP6V1A/GALC/ATP6V1B2/IL1B/RRAGD/LAMP2/ACP5/SDCBP/LYZ/VNN1 | |
| vacuole | 13 | GNS/GPR137B/CTSC/ATP6V1A/GALC/ATP6V1B2/IL1B/RRAGD/LAMP2/ACP5/SDCBP/LYZ/VNN1 | |
| cytoplasmic vesicle part | 19 | ITPR1/GNS/PKD2/ATP8B4/SNX8/FGR/CXCL1/CTSC/S100A8/IQGAP2/USO1/SNX10/PAM/LAMP2/C5AR1/SDCBP/LYZ/PNP/VNN1 | |
| primary lysosome | 6 | GNS/CTSC/LAMP2/SDCBP/LYZ/VNN1 | |
| azurophil granule | 6 | GNS/CTSC/LAMP2/SDCBP/LYZ/VNN1 | |
| cell leading edge | 9 | EPB41L3/APBB1IP/DOCK8/PKD2/FGR/IQGAP2/ATP6V1B2/LCP1/SAMSN1 | |
| cytoplasmic vesicle | 24 | ITPR1/GNS/PKD2/F2R/ATP8B4/EHBP1/SNX8/FGR/CXCL1/CTSC/S100A8/IQGAP2/USO1/ATP6V1B2/IGFBP2/SNX10/PAM/LAMP2/GZMB/C5AR1/SDCBP/LYZ/PNP/VNN1 | |
| **Molecular Function** |  |  | |
| actin filament binding | 5 | DSTN/IQGAP2/LCP1/CTNNAL1/TMOD1 | |
| calcium channel regulator activity | 3 | ITPR1/SGK1/TSPAN13 | |
| SH3/SH2 adaptor activity | 3 | SH2B3/CD28/SKAP2 | |
| cytoskeletal protein binding | 11 | ATF5/EPB41L3/DSTN/PKD2/S100A8/IQGAP2/LCP1/STK38L/SDCBP/CTNNAL1/TMOD1 | |
| actin binding | 7 | EPB41L3/DSTN/IQGAP2/LCP1/STK38L/CTNNAL1/TMOD1 | |
| calcium-release channel activity | 2 | ITPR1/PKD2 | |
| cytokine receptor binding | 5 | SH2B3/CCL4/CCL21/IL1B/SDCBP | |
| phosphatidylinositol binding | 5 | ITPR1/SNX8/IQGAP2/SNX10/SDCBP | |
| ligand-gated calcium channel activity | 2 | ITPR1/PKD2 | |
| phosphoprotein binding | 3 | PKD2/FGR/SAMSN1 | |
| **KEGG** |  |  | |
| Rheumatoid arthritis | 6 | CD28/CXCL1/ATP6V1A/ATP6V1B2/IL1B/ACP5 | |
| Lysosome | 5 | GNS/CTSC/GALC/LAMP2/ACP5 | |
| Graft-versus-host disease | 3 | CD28/IL1B/GZMB | |
| mTOR signaling pathway | 5 | DDIT4/SGK1/ATP6V1A/ATP6V1B2/RRAGD | |
| Type I diabetes mellitus | 3 | CD28/IL1B/GZMB | |
| Viral protein interaction with cytokine and cytokine receptor | 4 | CCL4/IL10RA/CXCL1/CCL21 | |
| NF-kappa B signaling pathway | 4 | CCL4/CXCL1/CCL21/IL1B | |
| Cortisol synthesis and secretion | 3 | ITPR1/NCEH1/PBX1 | |
| Oxidative phosphorylation | 4 | NDUFA4L2/COX7A1/ATP6V1A/ATP6V1B2 | |
| Epithelial cell signaling in Helicobacter pylori infection | 3 | CXCL1/ATP6V1A/ATP6V1B2 | |

Abbreviations: USCP, unstable carotid plaque; DEG, differentially expressed genes; KEGG, kyoto encyclopedia of genes and genomes.

**Additional Table S2.** Complete list of DEGs from three algorithms via CytoHubba plug-in.

| Degree | Betweenness | Closeness | Intersection |
| --- | --- | --- | --- |
| IL1B | IL1B | IL1B | IL1B |
| CCL4 | FGR | CCL4 | FGR |
| IL10RA | APBB1IP | IL10RA | APBB1IP |
| GZMB | GZMB | GZMB | GZMB |
| CD28 | LCP1 | FGR | LCP1 |
| CXCL1 | SKAP2 | CD28 | SKAP2 |
| FGR | CCL4 | CXCL1 | CCL4 |
| LCP1 | IL10RA | LCP1 | IL10RA |
| NKG7 | SNX10 | NKG7 | SNX10 |
| LYZ | CTSC | LYZ | CTSC |
| LAMP2 | NKG7 | S100A8 | NKG7 |
| SGK1 | CXCL1 | CCL21 | CXCL1 |
| CCL21 | LYZ | C5AR1 | LYZ |
| S100A8 | CD28 | SOD2 | CD28 |
| ATP6V1A | SGK1 | F2R | LAMP2 |
| RRAGD | LAMP2 | CEBPD | S100A8 |
| ATP6V1B2 | S100A8 | ACP5 | SAMSN1 |
| GPR183 | GPR137B | GIMAP4 | GPR183 |
| C5AR1 | NABP1 | GPR183 | C5AR1 |
| SNX10 | DDIT4 | APBB1IP | CCL21 |
| GIMAP4 | SAMSN1 | CTSC |  |
| CTSC | GPR183 | SAMSN1 |  |
| APBB1IP | DENND2D | DSTN |  |
| SKAP2 | ATP8B4 | SKAP2 |  |
| SOD2 | TSPAN2 | USO1 |  |
| GPR137B | PHOSPHO2 | SNX10 |  |
| NABP1 | DSTN | SNX8 |  |
| DDIT4 | C5AR1 | LAMP2 |  |
| SAMSN1 | F2R | ATP6V1A |  |
| DENND2D | CCL21 | RRAGD |  |

Abbreviations: DEG, differentially expressed genes.

**Additional Figure S1.** Soft thereshold selection and gene cluster tree via WGCNA of Uremia


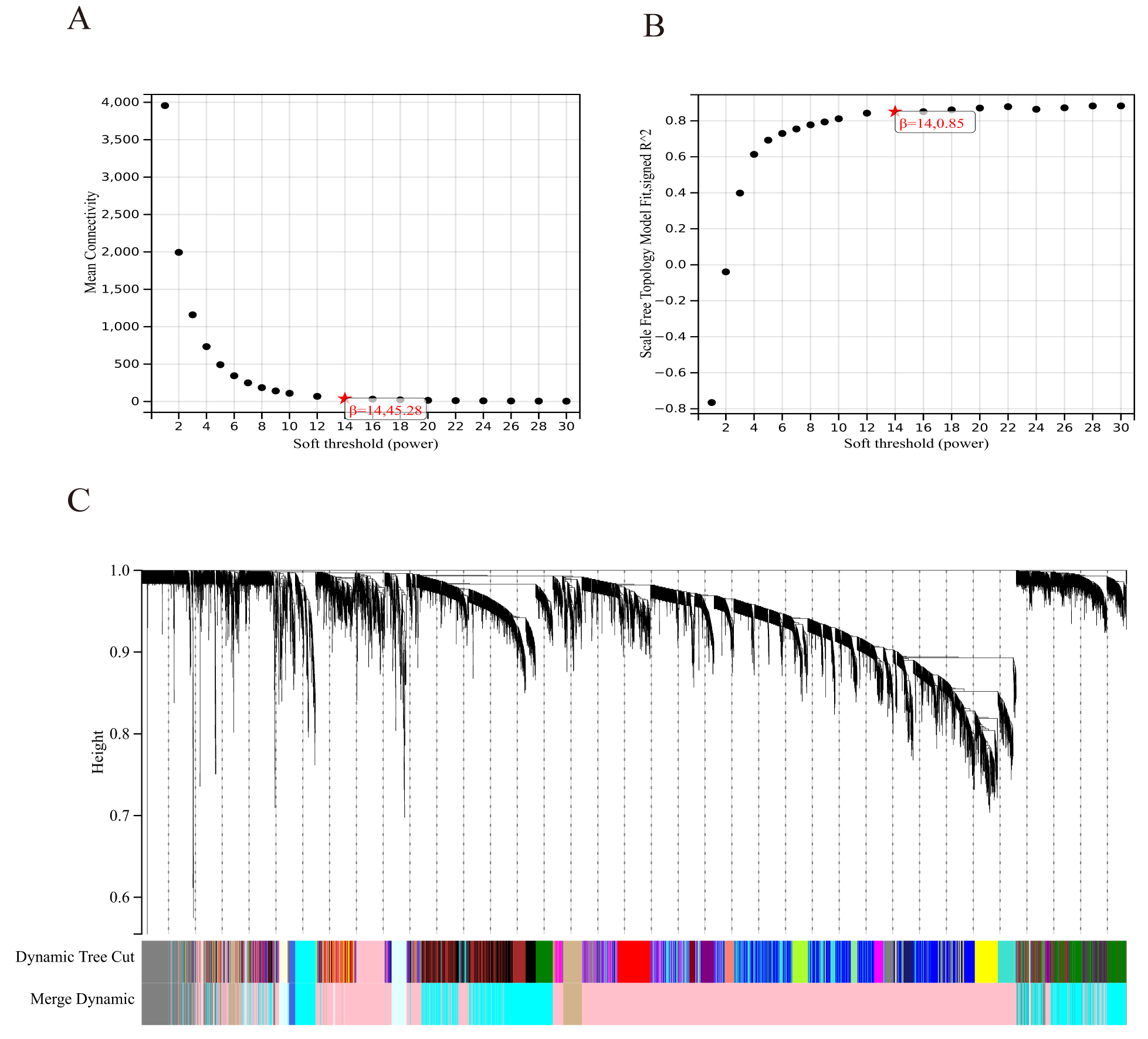


**(A-B)** The soft threshold selection. β=14 was chosen as the most appropriate threshold.

**(C)** Gene cluster tree of different modules.

Abbreviations: WGCNA, weighted gene co-expression network analysis.

**Additional Figure S2.** Soft thereshold selection and gene cluster tree via WGCNA of USCP


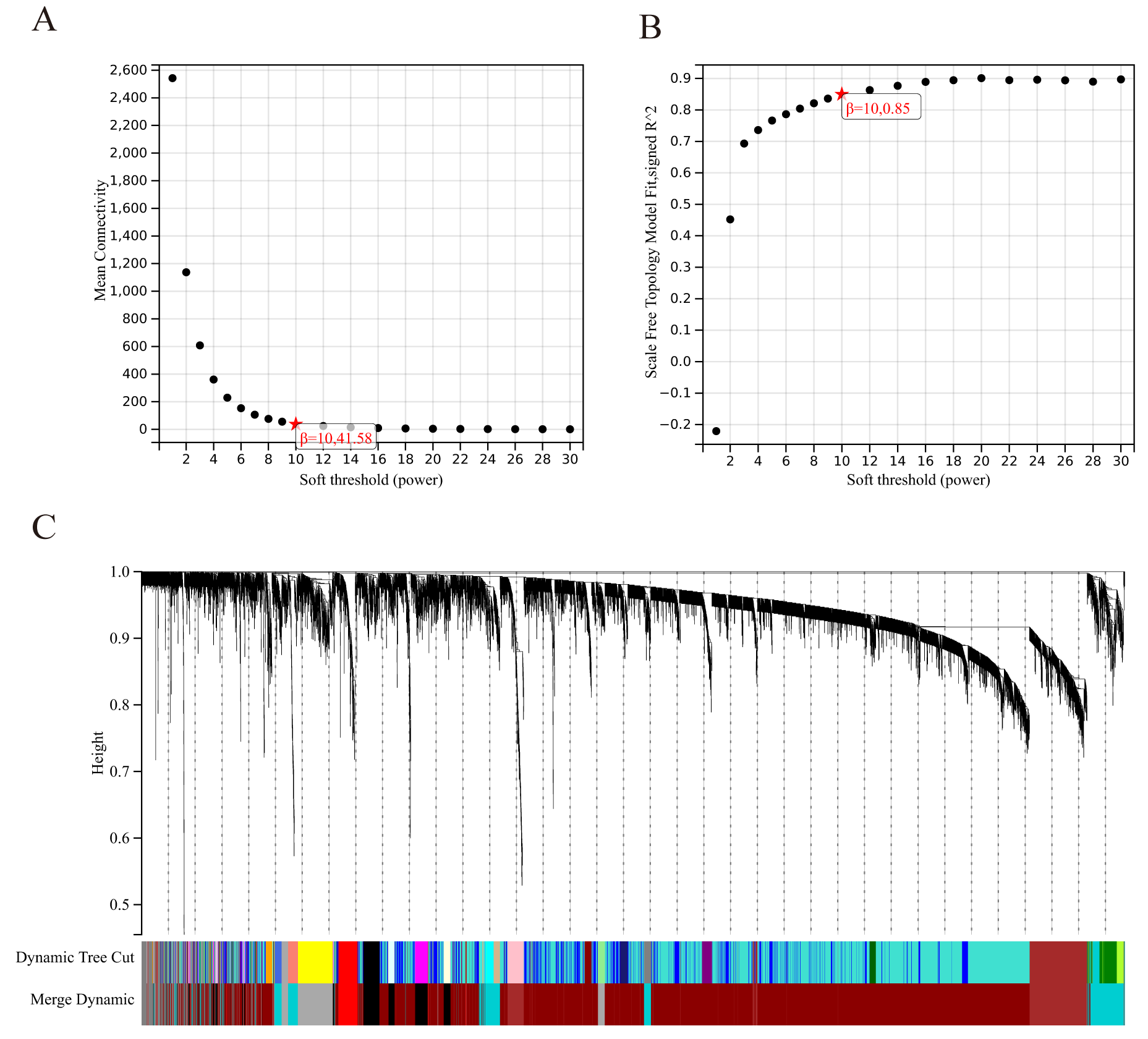


**(A-B)** soft threshold selection. β=10 was chosen as the most appropriate threshold.

**(C)** Gene cluster tree of different modules.

Abbreviations: WGCNA, weighted gene co-expression network analysis; USCP, unstable carotid plaque.
